# Impact of haptic feedback on applied intracorporeal forces using a novel surgical robotic system—a randomized cross-over study with novices in an experimental setup

**DOI:** 10.1007/s00464-020-07818-8

**Published:** 2020-07-22

**Authors:** Johanna Miller, Manuel Braun, Johannes Bilz, Sebastian Matich, Carsten Neupert, Wolfgang Kunert, Andreas Kirschniak

**Affiliations:** 1 Clinic of General, Visceral and Transplant Surgery, Working Group Surgical Technology and Training, Tübingen University Hospital, Waldhörnlestrasse 22, 72072 Tübingen, Germany; 2 Clinic for Orthopaedics, Tübingen University Hospital, Waldhörnlestrasse 22, 72072 Tübingen, Germany; 3 Department of Electromechanical Design, Darmstadt Technical University, Merckstrasse 25, 64283 Darmstadt, Germany

**Keywords:** Surgical robot, Haptics, Force feedback, Randomized controlled trial, Experiment setup

## Abstract

**Background:**

Most currently used surgical robots have no force feedback; the next generation displays forces visually. A novel single-port robotic surgical system called FLEXMIN has been developed. Through an outer diameter of 38 mm, two instruments are teleoperated from a surgeon’s control console including true haptic force feedback. One additional channel incorporates a telescope, another is free for special instrument functions.

**Methods:**

This randomized cross-over study analyzed the effect of haptic feedback on the application of intracorporeal forces. In a standardized experiment setup, the subjects had to draw circles with the surgical robot as gently as possible. The applied forces, the required time spans, and predefined error rates were measured.

**Results:**

Without haptic feedback, the maximum forces (median/IQR) were 6.43 N/2.96 N. With haptic feedback, the maximum forces were lower (3.57 N/1.94 N, *p* < 0.001). Also, the arithmetic means of the force progression (*p* < 0.001) and their standard deviations (*p* < 0.001) were lower. Not significant were the shorter durations and lower error rates. No sequence effect of force or duration was detected. No characteristic learning or fatigue curve was observed.

**Conclusions:**

In the experiment setup, the true haptic force feedback can reduce the applied intracorporeal robotic force to one-half when considering the aspects maximum, means, and standard deviation. Other test tasks are needed to validate the influence of force feedback on surgical efficiency and safety.

## Regaining the sense of touch

In a surgical environment, haptics plays an important role for tissue assessment and interaction. Surgeons mainly use tactile and force feedback to identify anatomical structures like vessels or nerves, to distinguish between healthy and diseased tissue as well as for instrument control. The role of haptic feedback in surgery is still widely discussed, especially since the introduction of robotically assisted procedures [1]. When using a master–slave setup, the instrument–tissue interaction point and the surgeon’s hand are physically disconnected. As a result, all conceivable haptic feedback is completely eliminated [2, 3]. Restoring the haptic information involves a three-step procedure: tactile or force assessment, digital signal transformation, and display as a perceivable feedback in the haptic interface. In general surgery, no generic haptic feedback is in clinical use to date. However, technical realization has already been proven in experimental setups [4, 5]. Possible causes are high demands made of the sensors such as sterilizability, insensitivity to influences within the abdominal cavity, and also the imitation of true haptic perception.

## “True haptic perception”

With this term we describe force feedback without changing the somatosensory modality. The opposite could be pseudo-haptic feedback, where visual and tactile vibrations are transmitted to symbolize grasping forces between the instrument jaws [6, 7]. To differentiate the sensor channels, we take a closer look at the tactile sense when describing a passive recognizing process of several different tactile stimuli. It includes static proprioception (spatial awareness), visceroception (visceral signaling), nociception (perception of pain), thermoception (perception of temperature differences),, and surface sensitivity (perception of mechanical irritations such as compression, vibration,, and elongation). Some tactile sensations are perceived with direct conjunction to kinaesthetic receptors [8]. The combination of passive tactile feedback (tissue perception) and dynamic kinaesthetic feedback (motion perception, as part of proprioception) is considered haptic feedback [9, 10]. Consequently, haptics generally stands for an active procedure of exploration and requires interaction with an object. It can be synonymously described as a “tentative understanding.” For the interpretation of all sensory information,, we make use of different exploratory procedures: unsupported holding, enclosure, applied pressure, lateral motion,, and contour following [11–13]. The pursued object properties to be determined by a surgeon are mainly size, surface, and material characteristics as well as position.

## Sensitive endoscopic surgeons

Minimally invasive surgeons usually draw conclusions regarding tissue texture by visually examining the tissue surface. However, with the exclusive use of visual feedback, the subsurface stays hidden. For detailed tissue assessment,, visual feedback has to be combined with kinaesthetic feedback in the course of dynamic examination. Tissue elasticity, for instance, can be estimated by observing the tissue deformation during force application [14]. Some studies have shown that providing both visual and kinaesthetic feedback is superior to solely visual or kinaesthetic feedback in terms of tissue characterization [15, 16]. Other working groups have argued that direct or indirect visual monitoring during tasks performed minimally invasively can completely compensate the absence of haptic feedback [17]. It is undeniable that additional visual information definitely leads to an improvement in a person’s motor skills [18]. However, the reliance on exclusively visual feedback attempts to make a virtue out of a necessity since no haptic feedback is available to date. The effect of reduced haptic feedback is also seen in laparoscopic surgery. Boer et. al [19] found that sensitivity feedback qualities of commercially available laparoscopic dissectors are eight times inferior to those of bare fingers. Besides the elimination of direct contact with the object (no tactile feedback), the transformation of applied forces through the instrument shaft is of special interest (modified kinaesthetic feedback) [3, 20, 21]. Force quality is influenced by inverted movement directions (fulcrum effect). Force quantity is affected by torsion forces in the abdominal wall and friction forces in the trocar [22].

## Experiment haptic setup

**Fig. 1:**
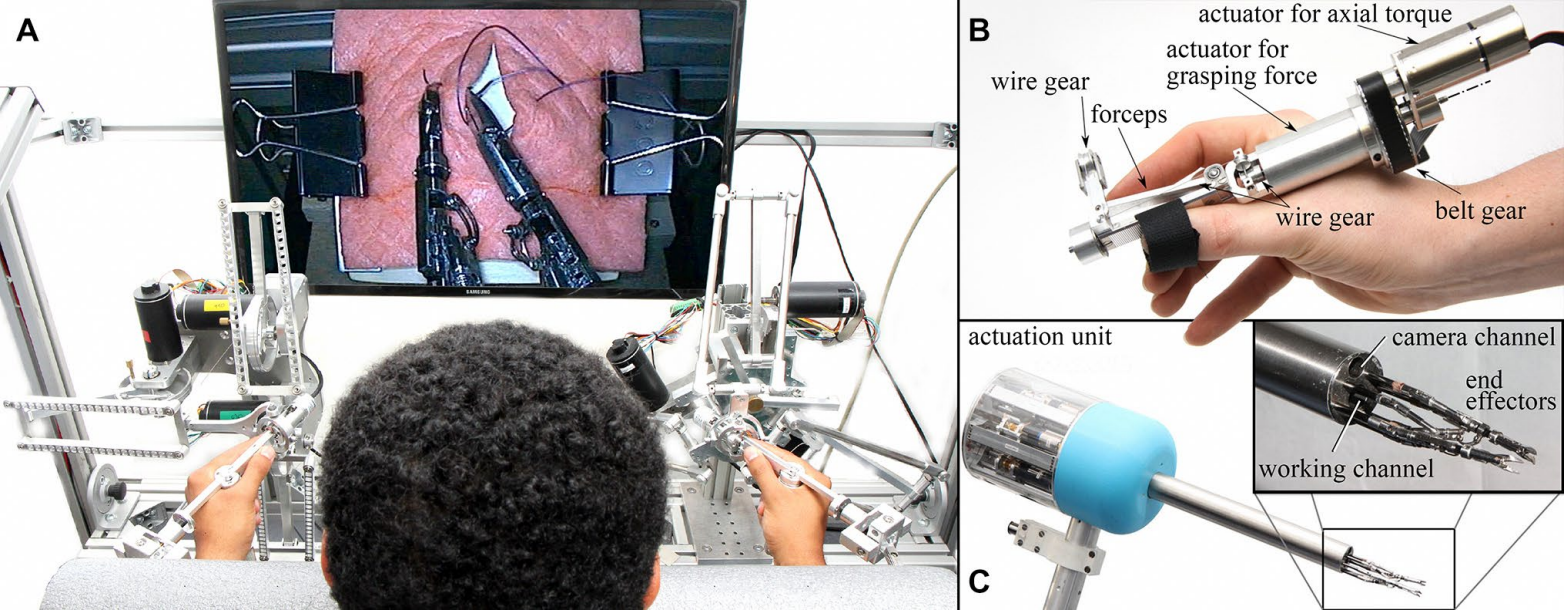
Single-port master–slave robotic system FLEXMIN. **A** Surgeon’s control console with haptic interface. **B** Haptic handle with force feedback. **C** Intracorporeal single-port instrument set with activation unit

To investigate the role of haptic feedback in robotic surgery, a novel telerobotic master–slave operating system, called FLEXMIN (Fig. 1) [23, 24], was developed in a cooperation between Tübingen University Hospital and Darmstadt Technical University. The aim and result of this R&D project funded by the German Research Foundation (DFG) was a single-port system that, in particular, can be inserted rectally as for transanal endoscopic micro-surgery (TEM/TEO [25]). It incorporates two instruments remote-controlled from a surgeon’s control console with force feedback. The slave robot with an outer diameter of 38 mm has a camera channel for a long telescope and a working channel for insertion of special assistance instruments (Fig. 1C).

## Research question

We still lack evidence whether haptic feedback gives additional information to the surgeon, which then helps improve surgical techniques and reduce perioperative complications. Consequently, the relevance of haptics in this context remains unclear [18]. The aim of the present study is to quantify the impact of tactile perception on applied forces using the surgical robot FLEXMIN in an experiment setup.

## Materials and methods

### Master-slave system FLEXMIN

The presented study was conducted with the FLEXMIN system (Fig. 1), a single-port surgical robot (outer shaft diameter 38 mm, two instruments with four degrees of freedom, Simulink real-time target machine, sampling rate 4 kHz, hardware connected via EtherCAT bus, bandwidth of force sensor ~ 6 kHz, and delay between force measurement and feedback (1 ms). The study subjects were operated the right FLEXMIN slave instrument with their right hand at the right haptic interface (Fig. 1B) [26, 27]. FLEXMIN’s control unit recorded the trajectories of the instrument tip and also its applied force with the help of an additional 6-axis force/torque (f/t) sensor (Nano17-E, ATI Industrial Automation, Apex, NC, USA). For live image presentation, a laparoscope (R. Wolf, 10 mm, 25° viewing direction, two-dimensional) and an endocam (R. Wolf, 3CCD, PAL) were used. The subjects read their task instructions on an LCD monitor on their right side and viewed the endovideo on a second LCD monitor (24″) in front of them.

### Study group

The study group included 31 subjects with no experience in laparoscopic or robotically assisted surgical interventions. Exclusion criteria were any movement disorder or other neurological disease conditions influencing the motor system of the upper body. Necessary precondition for inclusion was at least average motor coordination skills when completing the Purdue Pegboard Test [28]. Three subjects were left-handed, but not excluded because they scored sufficient results on the Purdue Pegboard Test using their non-dominant right hand. One student was not able to complete the test phase due to technical failure. All participants granted their written consent before joining the study. After enrollment, the subjects were randomly and equally allotted to two groups (Fig. 4).

### Standardized test task

**Fig. 2:**
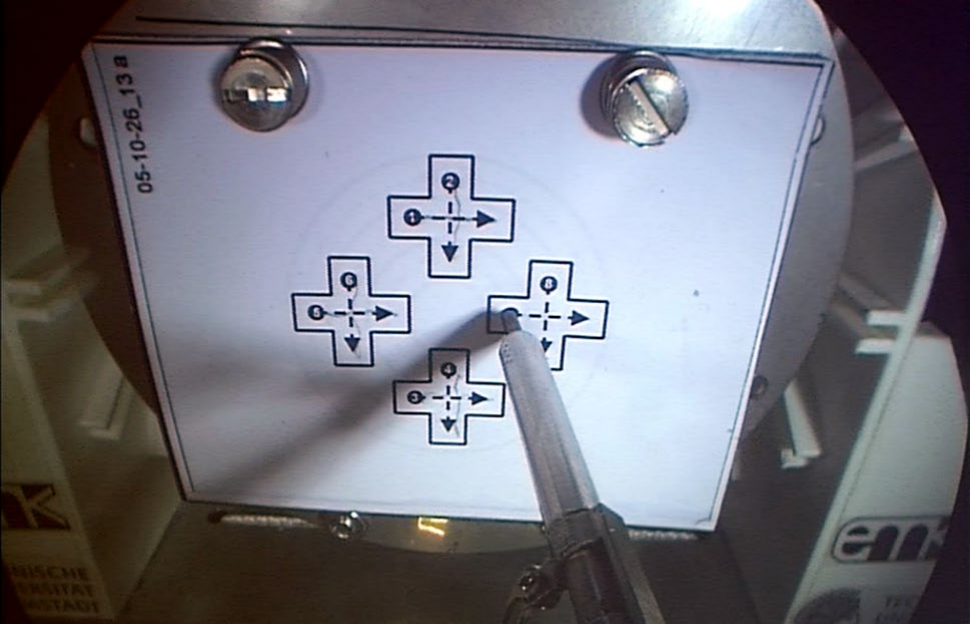
Training task

**Fig. 3:**
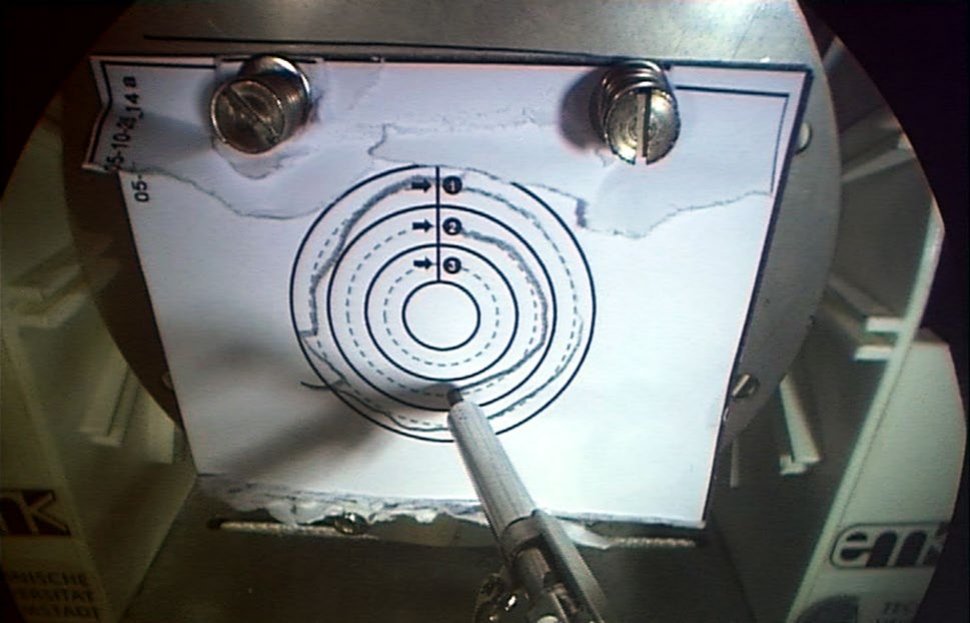
Test task

The test task called for precise positioning and moving of the right instrument tip. Paper sheets were vertically positioned in front of the master–slave console in systematic consideration of ergonomic posture. To ensure standardized conditions, each test round was conducted on a new paper sheet. For warm-up, a task with four equal crosses and straight dotted lines was used (training task, Fig. 2). The main task showed three circles of various size and drawn with a dotted line (test task, Fig. 3). Installed behind the sheets was the force/torque sensor for measurement of the applied touching force in XYZ direction. The aim was to continuously trace the dotted straight and concentric lines within predetermined limits, with minimal force and without lifting the pencil lead. The pencil lead was fixed to the right robot effector. Leaving the boundaries was counted as an X/Y error. Lifting the pencil lead off the paper sheet was counted as a Z error. The reason for choosing a planar circle to be drawn as the test task was to avoid several kinds of bias. Firstly, the task should be as easy as possible to understand in order to avoid information bias. Secondly, all subjects know how to drawn a circle on a flat paper. Therefore, there is no learning curve for the manual action itself. Thirdly, following the circular curve located in space requires continuous readjustment of movement in all three directions in space. Fourthly, grasping actions would involve an additional grasping force that would overlay the drawing force. Since both have to be transmitted with the same handle, movement and grasping forces might interfere with each other. The drawing action avoids such a bias. The drawing of crosses as a warm-up task was designed to be even more simple, while also requiring continuous gentle contact with the paper.

### Statistical endpoints

Primary statistical endpoint was the assessment of the applied maximum touching force. Our hypothesis was that applied forces are reduced when using haptic feedback. The time required for performance of each circle and the number of X/Y and Z errors were defined as second endpoints.

### Study design

**Fig. 4:**
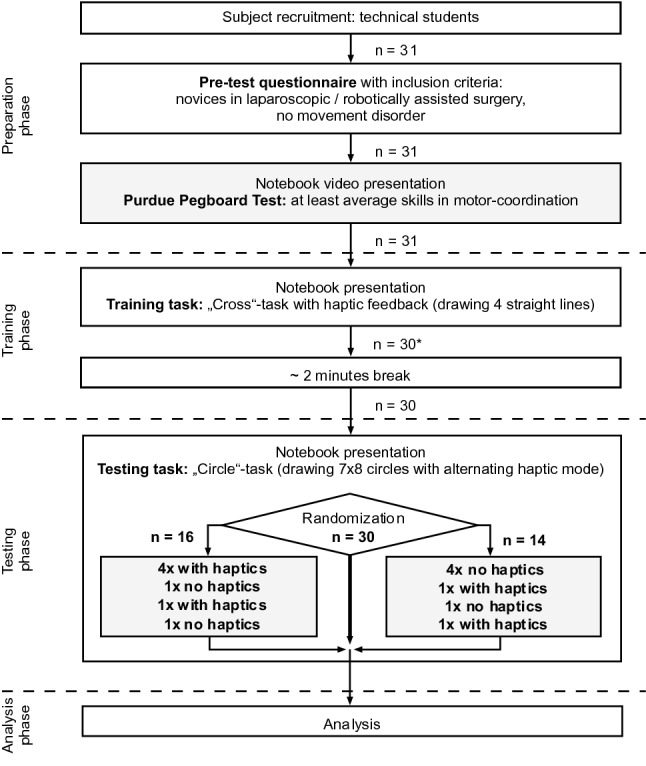
Study design. *The last subject #31 could not finish the training phase because the robotic system experienced a technical problem

The presented study was a two-armed prospective trial including 31 participants. The number of participants was identified by considering effect sizes. The diagram (Fig. 4) shows the study design as described below. All subjects completed a pre-test questionnaire asking for individual parameters such as age, sex, and handedness. Three subjects were left-handed and 28 were right-handed. With regard to time management and technical realization, students had to complete an abbreviated version of the Purdue Pegboard Test as instructed by a video and while referring to the Purdue Pegboard Test Manual. All instructions were shown as text on an additional monitor to the right of the endo monitor. The two tasks were demonstrated in brief videos shown on the same monitor. The first task was about picking up and placing items with the right hand (three times, 30 s each time). The second task was a bimanual assembly test procedure (three times, one minute each time). All 31 study participants showed at least average manual skills. For the purpose of familiarizing themselves with the master–slave console, a training phase followed. The training task instructions were demonstrated in a notebook presentation. The subjects were encouraged to trace four crosses consisting of eight straight dotted lines within given borders by operating the master–slave console with activated haptic feedback. Because of a technical failure on the first test day, that day’s last student was not able to complete the training task and was therefore excluded. After a 2-min break, the participants started the test phase. Under consideration of possible interindividual performance differences, a cross-over design was chosen for this phase. After another notebook presentation giving instructions for the test task, subjects were randomly divided into Group A (*n* = 16) and Group B (*n* = 14). The unequal group sizes were caused by the randomization combined with the unexpected end of the training phase. The test task was to trace three circular lines six times within predetermined limits (large external circle first, medium circle next, small inner circle last). Group A was started by performing four test repetitions with activated haptic feedback (+), followed by one round without (−) and the last round with haptic feedback (+). Group B had the opposite sequence (− − − − + −). Consequently, a total number of 30 × 6 × 3 = 540 circles were traced.

### Data acquisition and documentation

The applied touching forces were recorded with Matlab software (The MathWorks, Inc., Natick, MA, United States) using the f/t sensor. After the test task, X/Y errors in the form of drifting lines were counted by two independent experts. Z errors were recorded as force values of zero newtons and separately counted. Start and stop times for each circle were manually marked by pressing a digital switch and were also recorded with Matlab software. For additional documentation, performance of all test tasks was videoed and transferred to a PC/notebook via USB video grabber.

### Statistical analysis

For statistical analysis, SPSS version 25 (IBM, Armonk, NY, United States) software was used. The groups were analyzed for normal distribution using the Shapiro–Wilk W test. For normally distributed measured values, the arithmetic means and standard deviations were calculated. For distribution-free data, the median values and the interquartile range (IQR) were calculated. No carryover effect caused by rising repetition number was found. If the measuring data (forces and performance times) were not normally distributed, the Wilcoxon test was used to analyze the significance. Probability values of *p* < 0.05 were considered significant.

## Results

### Demographics of participants

Questions 1 to 3 of the pre-test questionnaire were asked for demographic details on the participants. The age of the 31 study participants ranged from 22 to 32 years (25.1 ± 2.2 years); 29 were males and two were female. Twenty-eight subjects were right-handed, and three were left-handed.

Mean body weight was 78.7 kg ± 16.1 kg, and mean height was 181 cm ± 7.9 cm.

The questionnaire was also asked for regular manual activities. Of all study participants, 38.7% play an instrument (9.7% keyboard instrument, 29% string instrument), 64.5% do handicrafts (such as painting, drawing or sculpting) in their leisure time, 38.7% regularly play skill games and 77.4% play computer games (51.6% more often than once a month, 25.8% even more often than once a week).

The parameters age, body weight, and height did not influence performance quality regarding force and time effort, either with or without haptic feedback.

### Perdue Pegboard test

**Fig. 5:**
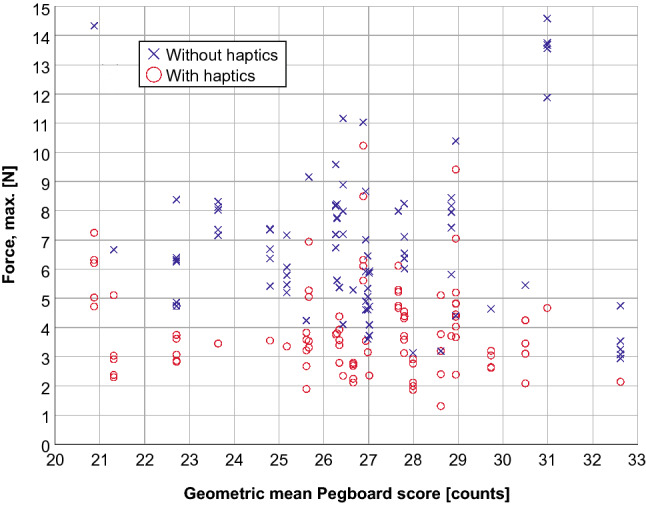
Applied maximum force [N] depending on manual dexterity represented by the Perdue Pegboard Test score [counts]

**Fig. 6:**
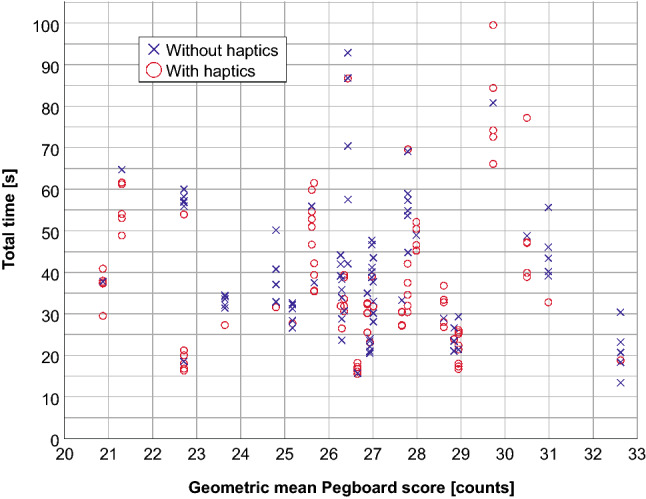
Total performance time [s] depending on Perdue Pegboard Test score [counts]

The results of the first Pegboard task (positioning with the right hand) do not allow any conclusions to be drawn regarding force or time expended by the subjects in the test phase, either with or without haptic feedback. The same applies for the bimanual assembly test. The maximum touching force (Fig. 5) and the total performance time (Fig. 6) depending on the geometric mean Pegboard score are illustrated below.

### First endpoint (touching force)

**Fig. 7:**
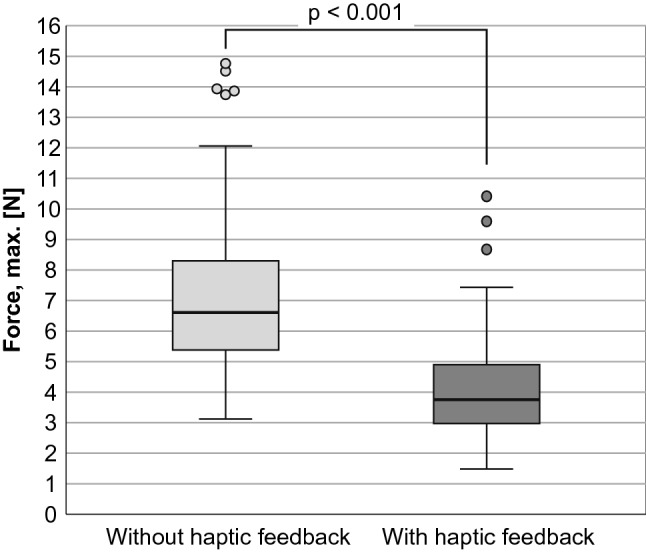
Maximum applied force [N] depending on haptic mode [*n* = 30]

**Fig. 8:**
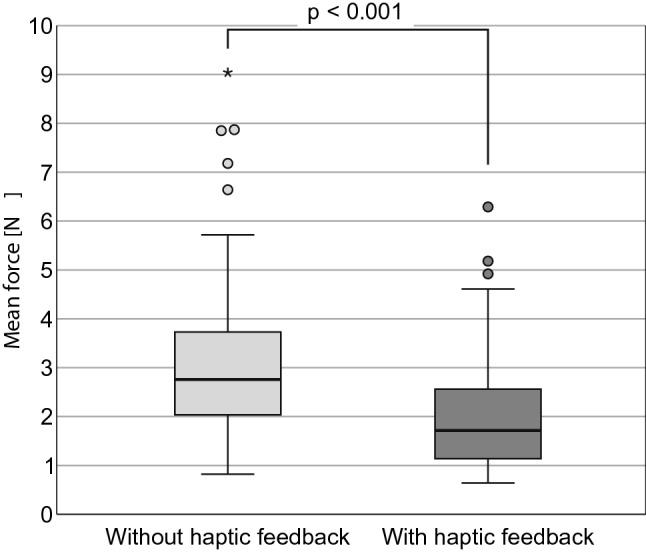
Mean applied force [N] depending on haptic mode [*n* = 30]

**Fig. 9:**
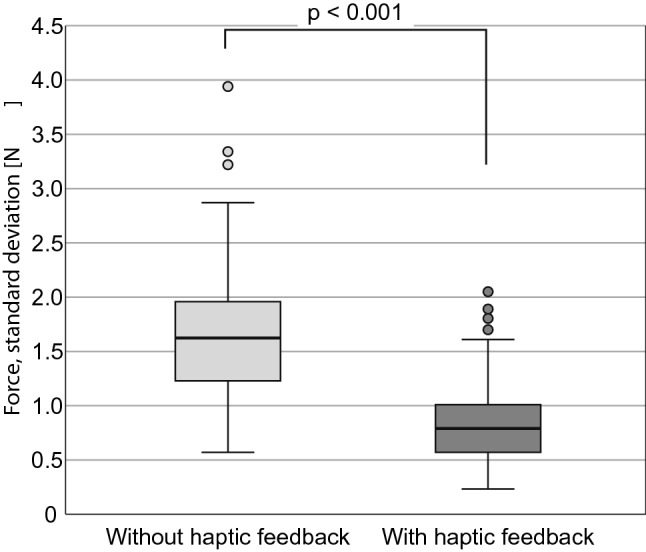
Standard deviation of applied forces [N] depending on haptic mode [*n* = 30]

**Fig. 10:**
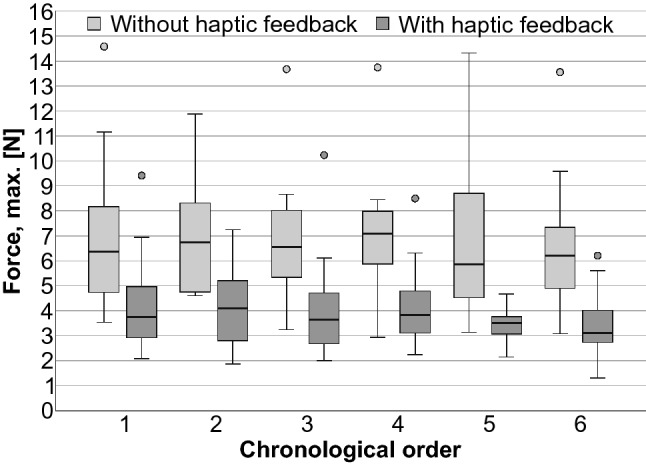
Maximum applied force [N] depending on haptic mode and chronological order [*n* = 30]

All measured data were distribution-free. Without haptic feedback, the maximum forces (median/min.–max./IQR) were 6.43 N/2.94 N–14.58 N/2.964 N. With haptic feedback, the maximum forces were significantly (*p* < 0.001) lower: 3.57 N/1.30 N–10.23 N/1.936 N (Fig. 7). When using haptic feedback, the mean forces also were significantly lower (*p* < 0.001) 1.72 N/0.64 N–6.29 N/1.42 N (Fig. 8), as well as the standard deviations of the force progressions (*p* < 0.001) 0.79 N/0.23 N–2.05 N/0.44 N (Fig. 9). No sequence effect was detected in the force (Fig. 10). No characteristic learning or fatigue curve was visible.

### Second endpoints

#### XY and Z errors

**Fig. 11:**
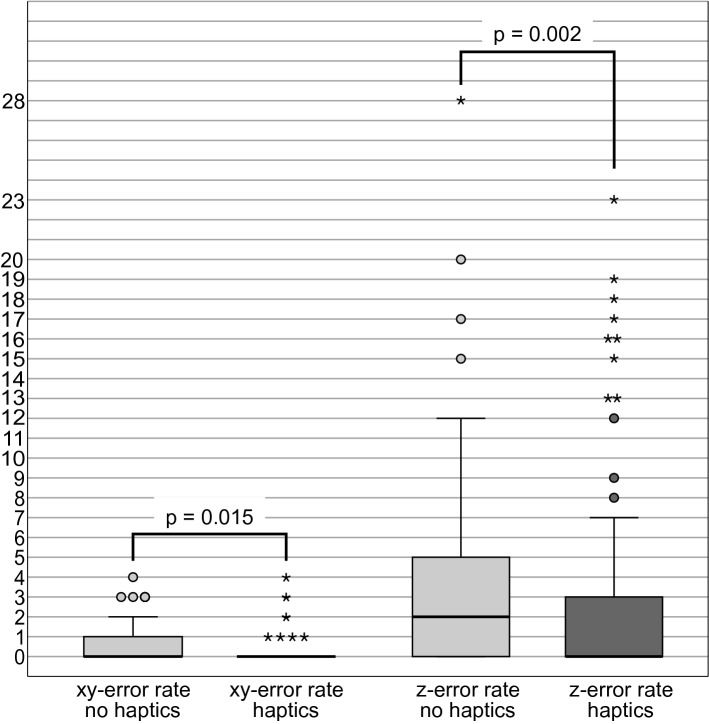
Error number depending on haptic mode [*n* = 30]

Similar to the first endpoint, no normal distribution was found for the second endpoints. The XY error rate (median/min.–max./IQR) without haptic feedback was 0/0–13.33%/3.33%. With haptic feedback, only the interquartile range differed and amounted to 0%. The Z error rate without haptics was 6.67/0–93.33%/16.67% versus 0/0–76.6%/7% with haptics. Analysis of both parameters showed no statistical significance (Fig. 11).

#### Performance time

**Fig. 12:**
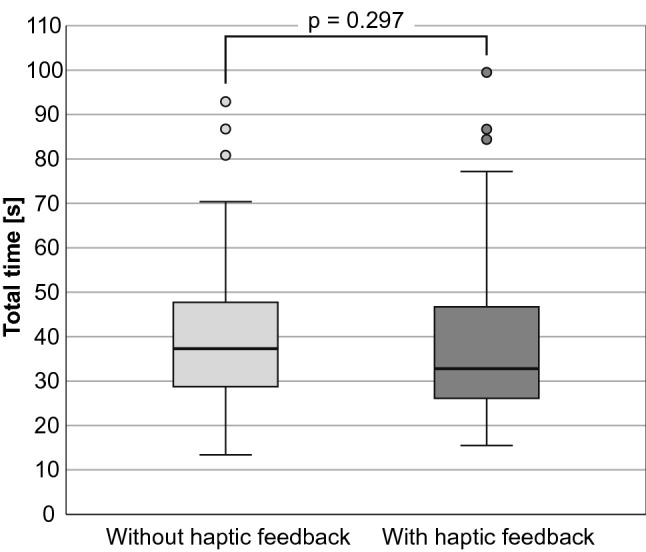
Total performance time [s] depending on haptic mode [*n* = 30]

Median performance time was minimally lower in the haptic group (32.8 s versus 37.3 s in the group without haptics), but without significance (Fig. 12). For the minimal and maximum performance time as well as the interquartile range, haptic feedback showed no positive influence.

## Discussion

**Fig. 13:**
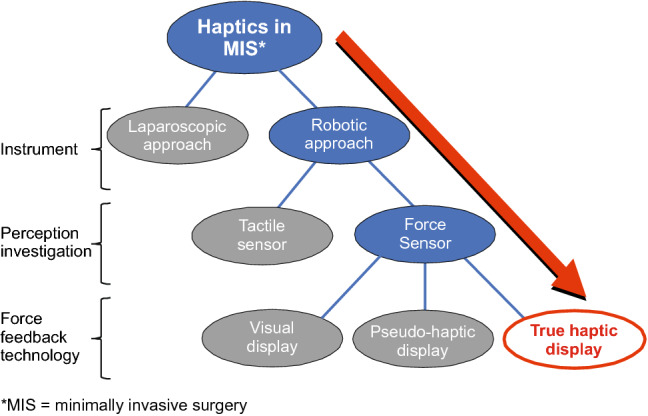
Classification of surgical haptics in the literature

The presented randomized comparative experiment trial showed a benefit of true haptic force feedback as indicated by a significant reduction in applied forces. In previous studies, maximum pulling forces were measured with the gall bladder in a box trainer [29]. The used traumatic graspers usually damaged the tissue when the pulling force exceeded a value of 23.3 N ± 8.2 N. In laparoscopy ,such brute forces are applied only with large graspers, namely to tissue that will be extracted. When using atraumatic graspers, the tissue slipped out at pulling forces in a range of 11.5 N ± 3.2 N, whereby it is not clear how “atraumatic” these manipulations really were. In the present study, maximum forces ranged from 2.9 to 14.6 N without haptics and from 1.30 to 10.23 N with haptics. Consequently, this reduction of the applied intracorporeal robotic forces by one-half could mean the difference whether a tissue is damaged or not. For better interpretation of our results, we classified the complex topic of haptics (Fig. 13) reported in the literature. Firstly, our study analyzed haptics in a robotic *not* involved in the laparoscopic surgical approach. Secondly, we measured only the three-dimensional application force at the tip of the surgical instrument, but *not* the tactile surface information. Therefore, we delivered force/kinesthetic feedback and *not* tactile/cutaneous feedback. Thirdly, the force feedback was conveyed as a true haptic force feedback unlike visual or pseudo-haptic representation [30].

Visual feedback can contain a lot of information, but is principally not transparent due to the implied change in sensation channel. There is a consensus concerning the fact that especially novices perform significantly faster and with fewer errors in box trainers or simulator studies when using three-dimensional (3D) laparoscopic imaging systems [31]. If we had used 3D vision in our study, like in the da Vinci robotic system, the non-haptics group might have benefitted more than the 2D group. However, the pencil lead cast a shadow on the paper, thus clearly indicating the moment of touch. In addition, the notepad was slightly elastic. Hence, applying a touch force resulted in visible movement of the paper surface. On the other hand, 3D vision requires adaption and selection of the subjects. For this reason, the authors preferred to use two-dimensional vision with helpful illumination instead of 3D. Most existing haptic displays emerged from virtual reality environments and generate only a pseudo-haptic feedback presented by vibrations or fluid surface variations. Only a few cases of true haptic force feedback are reported [32, 33]. However, until now, these concepts have not provided any proof of concept in general surgery. In future, haptic gloves might pose a new research area [34]. So far, also no surgical instrument has been clinically established that would allow imitation of the complex tactile movements between the fingertips as applied in open surgical procedures. Consequently, the only available tactile information is the three-dimensional instrument–tissue interaction force and possibly the grasping force [5]. This is why there is only little literature addressing surgical palpation with tactile sensors. Schostek et al. [35] technically described a tactile sensor with 3 × 10 tactile elements inside the instrument jaws. Perri et al. [36] evaluated a laparoscopic tactile sensing system with 4 × 15 tactile elements grasping ex vivo bovine liver tissue. The force distribution was shown visually in the form of a color contour pressure map and the total force in a bar chart. Perri found a 71% reduction in the applied maximum pressure as compared to an endoscopic grasper. However, his results are not comparable with ours, because the force was assessed between the jaws and not as a forward-pushing force exerted by the instrument tip. In addition, the feedback was displayed visually. Reiley et al. [37] hypothesized that visualization of applied forces is a haptic feedback surrogate. In his study, four surgeons with da Vinci experience and six novices completed a standardized knot tying task under different sensory substitution (no feedback, visual feedback, auditory feedback, combined audio-visual feedback). The applied force parameters were recorded by an instrument tracking system that was integrated in the da Vinci robot. The authors found that any sensory substitution scenario, alone or in combination, led to a measurable reduction of applied forces. However, the visual force feedback showed an advantage only for surgeons with no da Vinci training. The authors suspected that experienced surgeons are probably more adept at using visual cues for task completion [31]. Meccariello et al. [17] share the opinion that surgical experience can compensate the lack of haptic feedback. In an experimental palpation task, three membranes each with a different thickness had to be touched and then ordered according to hardness. In addition, a hidden metallic clip behind the membranes had to be identified. The expert surgeons scored 8.87 and significantly (*p* < 0.05) better than did the non-experts with a score of 3.57. They also logged a shorter performance time of 28.8 s compared to 71.3 s. Since only novices participated in our study, it can be assumed that if experts had participated we might have found less benefit from the haptic feedback, because the experts would have found more visual cues for force control. However, the “expert effect” can not be carried over to our study, because the tasks and the scoring were quite different [32]. Haouchine et al. [38] presented an approach to indirect estimation of instrument–tissue interaction forces without any mechanical force sensor. A stereoscopic camera gathered two slightly different perspectives that were used to calculate a three-dimensional biomechanical model on-the-fly. The interaction force was estimated from the computed tissue deformations and was visualized as an augmented live endovideo. Nevertheless, the system requires detailed surface textures and, difficult to access, subsurface elastic tissue properties. Sutherland et al. [32] designed an image-guided robot arm, called “neuroArm,” that allows the visual presentation of positional and instrument–tissue force information. It was used to resect glioma in 18 patients. The elaborate system is specialized for neurosurgery offering micro-manipulation, high sensitivity, and MR compatibility. The two-armed robot is designed for use in an open operative site and supported by additional manual instruments. Nevertheless, even though the robot is not suitable for the laparoscopic approach, the haptic console is of special interest. The applied forces were about 10 times smaller than those applied in our laparoscopic scenario. By the way, the “neuroArm” robot and also a force-sensing surgical tool [39] use the same kind of force sensor that we applied in our study. In contrast to the aforementioned works, studies have shown that the absence of haptic feedback can lead to excessive and inadequate force application [26]. In this context, a larger number of incidents of tissue damage [40] or inappropriate suture handling [4] have been described. In our comparative study, task performance was subjected to neither a learning nor a fatigue curve. It can be assumed that the test task was brief enough to not impair the subjects’ attention level. The highly significant results relativize a possible bias caused by the fact that the number of subjects in the two study arms differed slightly. Moreover, it seems that the handling of the FLEXMIN system was intuitive enough to perform the task straight away without a learning effect bias. This is the advantage of the standardized experimental setup. We are aware that it remains to be proven whether and how the impact of real haptic feedback can be transferred to clinical routine. So far, the FLEXMIN robotic system is a scientific tool and not approved for clinical use.

## Conclusion

In the experiment setup, the true haptic force feedback can reduce the applied intracorporeal robotic force by one-half considering the aspects maximum, mean, and standard deviation. To ensure smooth operation with insensitive surgical robots, the surgeon must have learned to interpret visual cues of force application. This is possible for experienced robotic surgeons as opposed to the novices in our study, but is certainly not intuitive. It is desirable to restore force sensation for the robotically operating physician. Giving the robotic surgeon force feedback is sensible in any case, especially since haptics is natural and perfectly compatible with diligent visual observation. Further studies with different test tasks and subjects having varying surgical experience are needed to validate the influence of force feedback and pseudo-haptic feedback on surgical efficiency and safety.
